# Differentiation Between Granulomatous Lobular Mastitis and Breast Cancer Using Quantitative Parameters on Contrast-Enhanced Ultrasound

**DOI:** 10.3389/fonc.2022.876487

**Published:** 2022-07-15

**Authors:** Liang Yin, Enock Adjei Agyekum, Qing Zhang, Lei Pan, Ting Wu, Xiudi Xiao, Xiao-qin Qian

**Affiliations:** ^1^ Department of Breast Surgery, Jiangsu University Affiliated People’s Hospital, Zhenjiang, China; ^2^ Department of Ultrasound, Jiangsu University Affiliated People’s Hospital, Zhenjiang, China; ^3^ Department of Pathology, Jiangsu University Affiliated People’s Hospital, Zhenjiang, China

**Keywords:** granulomatous mastitis, breast cancer, quantitative parameters, imaging characteristics, contrasted-enhanced ultrasound

## Abstract

**Objective:**

To investigate the Contrast-enhanced ultrasound (CEUS) imaging characteristics of granulomatous lobular mastitis (GLM) and the value of differentiating GLM from breast cancer.

**Materials and methods:**

The study included 30 women with GLM (mean age 36.7 ± 5 years [SD]) and 58 women with breast cancer (mean age 48. ± 8 years [SD]) who were scheduled for ultrasound-guided tissue biopsy. All patients were evaluated with conventional US and CEUS prior to the biopsy. In both groups, the parameters of the quantitative and qualitative analysis of the CEUS were recorded and compared. The receiver-operating-characteristics curves (ROC) were created. Sensitivity, specificity, cut-off, and area under the curve (AUC) values were calculated.

**Results:**

TTP values in GLM were statistically higher than in breast cancer (mean, 27.63 ± 7.29 vs. 20.10 ± 6.11), but WIS values were lower (mean, 0.16 ± 0.05 vs. 0.28 ± 0.17). Rich vascularity was discovered in 54.45% of breast cancer patients, but only 30.00% of GLM patients had rich vascularity. The AUC for the ROC test was 0.791 and 0.807, respectively. The optimal cut-off value for TTP was 24.5s, and the WIS cut-off value was 0.185dB/s, yielding 73.33% sensitivity, 84.48% specificity, and 86.21% sensitivity, 70% specificity respectively in the diagnosis of GLM. The lesion scores reduced from 4 to 3 with the addition of CEUS for the patients with GLM. However, the scores did not change for the patients with breast cancer.

**Conclusion:**

CEUS could help distinguish GLM from breast cancer by detecting higher TTP and WIS values, potentially influencing clinical decision-making for additional biopsies.

## Introduction

Granulomatous lobular mastitis (GLM) is a rare, benign chronic inflammatory disease with unclear etiology that is frequently mistaken as a malignant process both clinically and radiographically (1) Imaging features suggestive of GLM remain nonspecific and are not always present in all patients. As a result, accurate diagnosis necessitates pathological examination (2, 3). Unnecessary biopsies or surgical excision can result in chronic fistulas and breast deformities. During the evaluation, the patient may experience a great deal of anxiety.

Ultrasonography (US) is frequently used to assess lesions using Breast Imaging Reporting and Data System (BI-RADS), because it has a high sensitivity in diagnosing breast lesions (4). BI-RADS 3 lesions are likely to be benign and should be monitored; BI-RADS 4 and 5 lesions are suspected malignant and must be pathologically confirmed. Contrast-enhanced ultrasound (CEUS) imaging with microbubble contrast agents has significantly improved microcirculation visualization and allowed researchers to overcome the limitations of traditional B-mode US techniques. Previous studies have shown that combining CEUS with conventional US could improve diagnostic performance in breast lesions. The kinetic parameters of tumor tissue in CEUS can also be quantified by generating the time-intensity (T/I) curve with specialized software. Several studies have shown that CEUS can help distinguish between benign and malignant breast lesions (5–7). Because the current imaging modalities are not sufficient to establish a definitive diagnosis of GLM in most patients. Prior studies of breast cancer reveal increased amounts of microvessels in cancerous lesions.Based on this data, CEUS may provide additional information for distinguishing GLM from breast cancer lesions.

The purpose of this study was to investigate CEUS parameters of GLM and breast cancer and analyze their values in distinguishing GLM from breast cancer.

## Materials and Methods

### Study Design

The ethics committee of Jiangsu University Affiliated People’s Hospital approved this study (K-20190175-W), and written informed consent for breast CEUS examination was obtained prior to enrolling a patient in our study. Between September 2019 and November 2021, consecutive patients were screened for breast cancer at Jiangsu University Affiliated People’s Hospital’s Department of Breast Surgery. According to the BI-RADS classification scheme, the selection criteria were represented by US findings classified as BI-RADS category 3–5. All those patients contraindicated for CEUS were excluded from the study. After grayscale and contrast-enhanced ultrasound, a core needle biopsy (CNB) or vacuum-assisted biopsy (VAB) of BI-RADS 4 and 5 lesions was performed. Only the most suspicious lesion fulfilling the selection criteria was evaluated when a patient had multiple lesions. GLM diagnosis criteria was based on the management of granulomatous lobular mastitis: an international multidisciplinary consensus (2021 edition) (8). The histopathologic results of biopsy served as the diagnosis gold standard in this study. All patients had standard mammography and magnetic resonance imaging (MRI) (according to age).

### Ultrasound Equipment

All examinations were performed with a Philips EPI Q5 color Doppler ultrasound equipped with a high-frequency linear array probe (using a 12-5 MHZ and 9-3 MHZ linear-array transducer) and dedicated contrast pulse sequences. To reduce contrast agent destruction, low mechanical index values (MI=0.08) were used. The contrast medium employed was SonoVue (Bracco Imaging, Milan, Italy).

### Ultrasound Examination

The same sonologist, with 20 years of experience with breast US, performed all US and CEUS examinations. When a breast lesion was discovered, its location, maximum diameter, 2-D characteristics, and color Doppler characteristics were all recorded. Shape, margin, orientation, inner echo, posterior echo, and calcification were all 2-D characteristics. A dual display of grayscale and contrast-enhanced images was used to allow simultaneous visualization to keep the probe position constant during the examination. The plane with the most significant lesion diameter was chosen as the reference scan. In addition to keeping the transducer in a stable position throughout the scan, the target area was compressed as little as possible. The contrast reagent suspension used consisted of 59 mg of SonoVue powder mixed with 5 mL of saline and was administered *via* a 20-gauge cannula into the antecubital vein. Following a bolus injection of 4.8 mL of contrast agent *via* the intravenous cannula, a saline injection of 5–10 mL was administered. A two-minute dynamic image image was recorded and saved on a hard disk as raw data for later analysis.

### Image Analysis

Two other investigators who had not performed the conventional US and CEUS examinations and were blinded to surgical, histopathologic, and other imaging findings independently analyzed the US imaging data. The findings of the conventional ultrasound examination were evaluated using a standardized BI -RADS™ (Breast Imaging Reporting and Data System) for breast ultrasound. The diagnostic criteria for CEUS, according to a previously published study (5).

**Figure 1:**
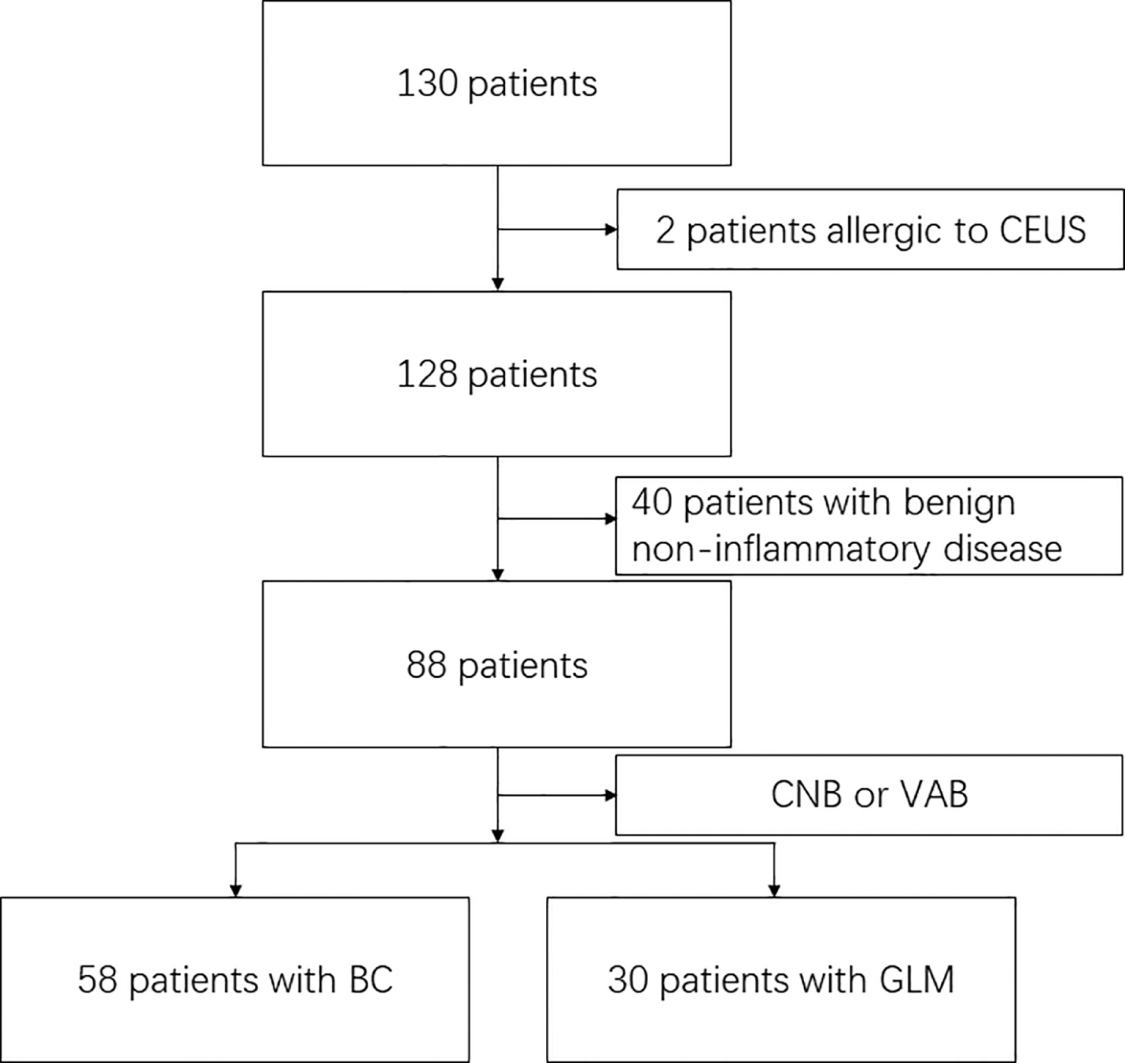
Flow chart with patients in the study. BC: breast cancer, granulomatous lobular mastitis : GLM; CEUS: contrast-enhanced ultrasound; CNB: Core needle biopsy; VAB: vcauum-assisted biopsy.

**Figure 2:**
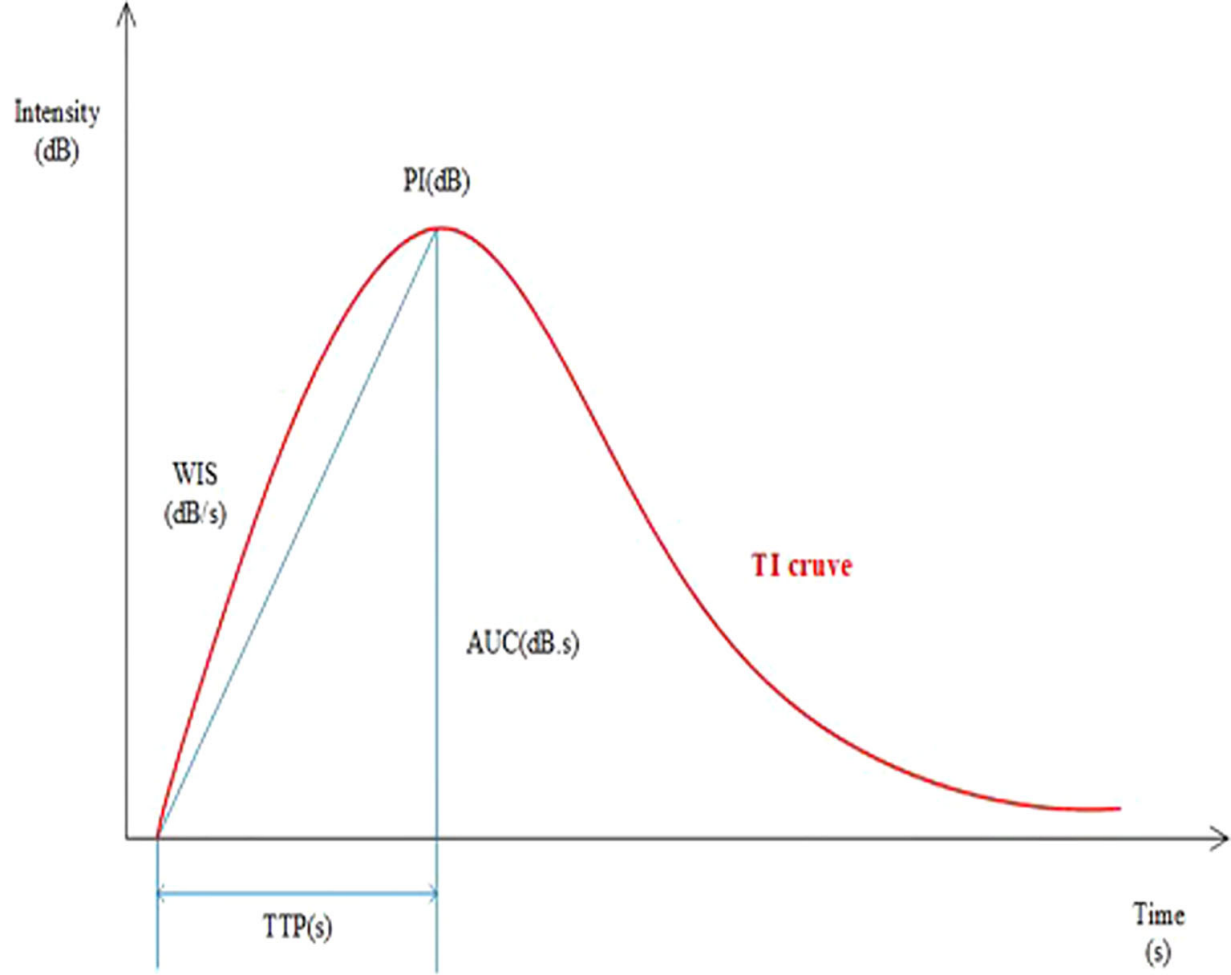
Representation of the time-intensity curve including parameters: TI (time intensity curves), PI (peakintensity), WIS (wash in slope), AUC (area under curve), TTP (time to peak).

A dedicated sonographic quantification software (Qontrast, Bracco, Milan, Italy) based on signal intensity pixel by pixel over time was used to generate color-coded maps of the studied lesion’s perfusion parameters. The enhancement patterns were evaluated as qualitative parameters, while the time-intensity curve was analyzed quantitatively (9). The qualitative variables were classified as follows: The degree of enhancement of lesions in comparison to surrounding tissue, the type of vascularization (peripheral or central), the homogeneity of perfusion (homogeneous vs. heterogeneous), and the degree of vascularization (peripheral or central) (weak or absent vs. intermediate vs. rich). The time-intensity curve’s quantitative parameters were determined. The region of interest (ROI) was placed in the area of most significant enhancement, and its size was set to the default value of 3 (mean, 6.9 ± 0.3 mm^2^; range, 5.6-7.5 mm^2^). The quantitative parameters were classified as follows: TTP (time to peak, s), PI (peak intensity, dB), WIS (wash in slope, dB/s), AUC (area under curve, dB x s). The integral value of the curve is associated with total blood volume and the sum of the area wash-in and area wash-out (**Figure 1**). Predefined motion compensation and background set were also applied to obtain these parameters. Motion compensation is an automatic function that detects slight movements in concordance with movements of ROI and eliminates their influence. Application examples are given (**Figure 2**).

### Statistical Analysis

For the statistical analysis, GraphPad Prism 7 was used. The student’s t-test and ANOVA were used to examine the differences between independent groups. The Mann-Whitney U test was used to compare differences between two independent groups. Fisher’s exact test was used to compare categorical (qualitative) parameters summarized using absolute and relative frequencies. The p< 0.05 level was considered statistically significant.

## Results

### Patients and Lesions Characteristics

**Figure 3:**
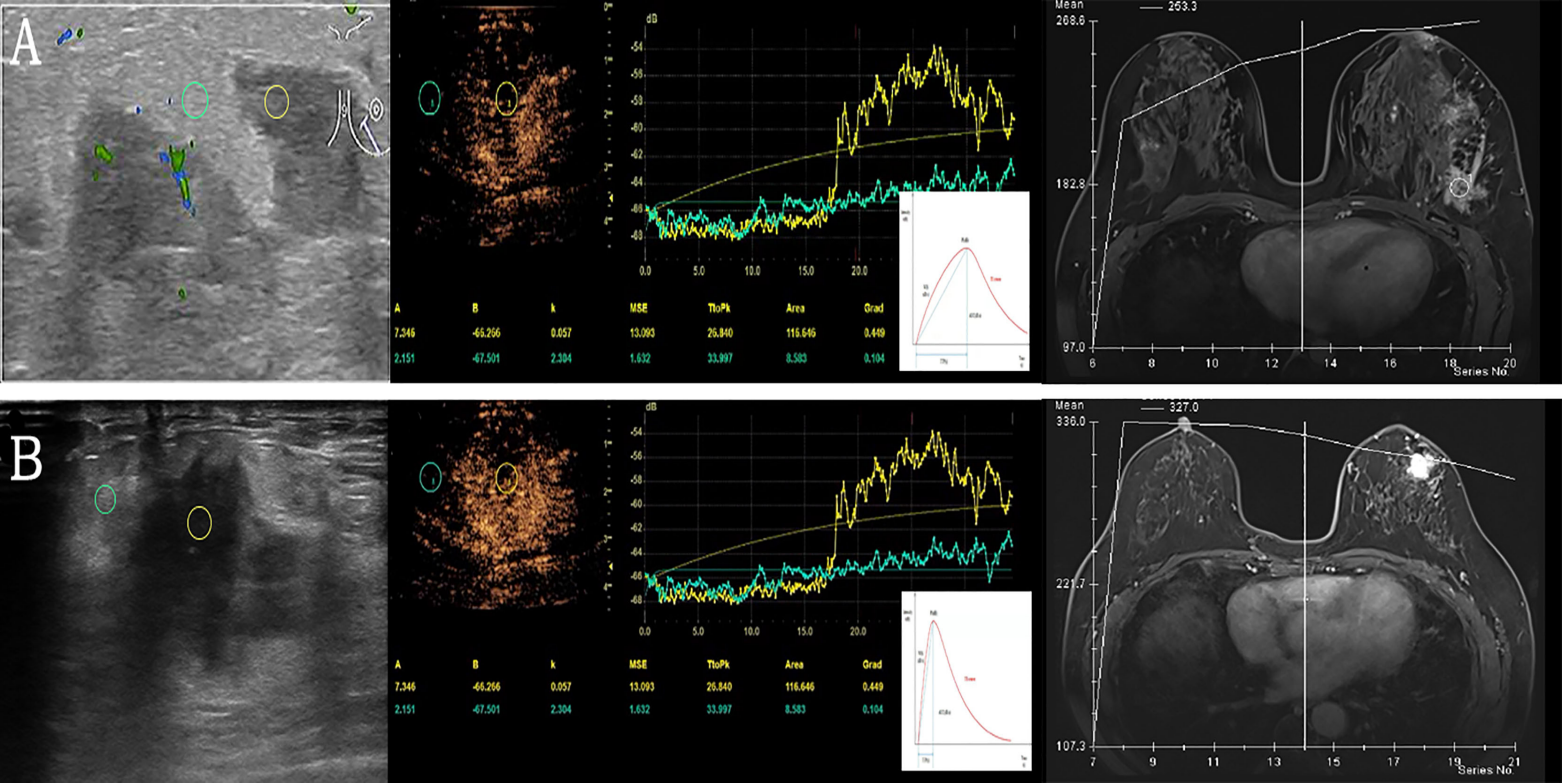
Examples of quantitative data acquisition using ROI. **(A)** Granulomatous lobular mastitis in a 28-year-old female. Moderate enhancement of the lesion (yellow ROI) compared to minimal enhancement in surrounding breast tissue (green ROI). Gradual enhancement and a gradual wash out of contrast agent (corresponding TIC below) versus TIC of DEC-MRI. **(B)** Invasive ductal carcinoma in a 61-year-old female with significant enhancement of the lesion (yellow ROI) compared to slight enhancement in surrounding breast tissue (green ROI), the lesion is ill-defined. After rapid enhancement of the tumor, early wash-out can be observed (corresponding TIC below) versus TIC of DEC-MRI. ROI, region of interest; TIC, time intensity curves; DEC-MRI, dynamic contrast enhancement magnetic resonance imaging.

A total of 130 patients who underwent image-guided biopsy were enrolled in this study. 2 patients were contraindicated for CEUS, and 40 patients diagnosed with the benign non-inflammatory disease (fibroadenoma, for example) were excluded. Fifty-eight patients with breast cancer and 30 patients with GLM were included in the analysis of the quantitative CEUS parameters (**Figure 3**).

Basic patient and lesion characteristics are summarized in **Table 1**. Patients with GLM were significantly younger (mean 36.7 ± 5 years vs. 48 ± 8 years) and had larger lesions (40.67 ± 8.38 vs. 29.02 ± 6.05 mm) (p< 0.01). There was no statistically significant difference in US-BI-RADS scores between breast cancer patients and GLM patients (p = 0.19). When only conventional US was used to evaluate patients with GLM, the scores were predominantly determined to be four according to the BI-RADS classification, similar to breast cancer, preventing differentiation. With the addition of CEUS for patients with GLM, the lesion scores decreased from 4 to 3. The scores for patients with breast cancer, on the other hand, remained unchanged.

**Table 1:** Basic patient and lesion characteristics.

	GLM	Breast cancer	p-Value
Lesions, n (%)	30 (52,6%)	58 (72.4%)	
Age	36.7 ± 5	48 ± 8	<0.0001
Symptoms			
Palpable mass	28	50	
Breast pain	30	12	
Erythema	24	1	
Nipple change	21	4	
Abscess	6	–	
Lesion localization			
Right (%)	20 (66.7%)	30 (51.7%)	0.26
Left (%)	10 (33.3%)	28 (48.3%)	
Lesion size (mm) (mean)	40.67 ± 8.38	29.02 ± 6.05	<0.0001
TIC			
I	21 (70.0%)	8 (13.8%)	<0.0001
II or III	9 (30.0%)	50 (86.2%)	
US-BI-RADS score (mean)	4.00 ± 0.52	3.88 ± 0.33	0.19
CEUS-BI-RADS score (mean)	3.20 ± 0.41	4.08 ± 0.28	<0.0001
Tumor Grade			
1	–	4 (6.9%)	
2	–	30 (51.7%)	
3	–	24 (41.4%)	
Histopathological type	–	n = 58	
invasive carcinoma NST	–	42 (72.4%)	
invasive lobular carcinoma	–	7 (12.1%)	
others	–	9 (15.5%)	

CEUS, contrast-enhanced ultrasound; TIC, time intensity curves; BI-RADS, Breast Imaging Reporting and Data System; NST, invasive carcinoma of no special type.

### CEUS Parameters in GLM and Breast Cancer

The parameters of the quantitative and qualitative analysis of the CEUS of GLM and breast cancer are summarized in **Table 2**. GLM had statistically higher TTP values (on average by 7 s) and lower WIS values (on average by 0.12 dB/s) than breast cancer. A statistically significant difference in the degree of enhancement was observed when rich vascularity was detected in 54.45% of breast cancer but only 30.00% of benign lesions. There was no discernible difference in the nature of the blood supply in the surrounding tissue of GLM and breast cancer.

**Table 2:** Quantitative and qualitative parameters of breast CEUS according to the diease.

	GLM	Breast cancer	*p*-value
Quantitative parameters			
TTP (s)	27.63±7.29	20.10 ± 6.11	<0.0001
WIS (dB/s)	0.16 ± 0.05	0.28 ± 0.17	<0.0001
PI (dB)	2.85 ± 0.91	2.91 ± 0.91	0.756
Qualitative parameters			
Type of vascularization			0.202
peripheral	25 (83.33%)	40 (68.97%)	
peripheral + central	5 (16.67%)	18 (31.30%)	
Perfusion homogeneity			
homogeneous	4 (14.81%)	3 (5.17%)	0.201
heterogeneous	23 (85.19%)	55 (94.83%)	
Perfusion homogeneity			
Enhancement degree			
poor/absent	2 (6.67%)	3 (5.17%)	0.009
intermediate	19 (63.33%)	14 (24.14%)	
rich	9 (30.00%)	31 (54.45%)	

CEUS, contrast-enhanced ultrasound; TIC, time intensity curves; TTP, time to peak; WIS, wash in slope; PI peak intensity; AUC, area under curve.

### CEUS-BI-RADS Categories, TTP and WIS Values of Patients With GLM

**Figure 4:**
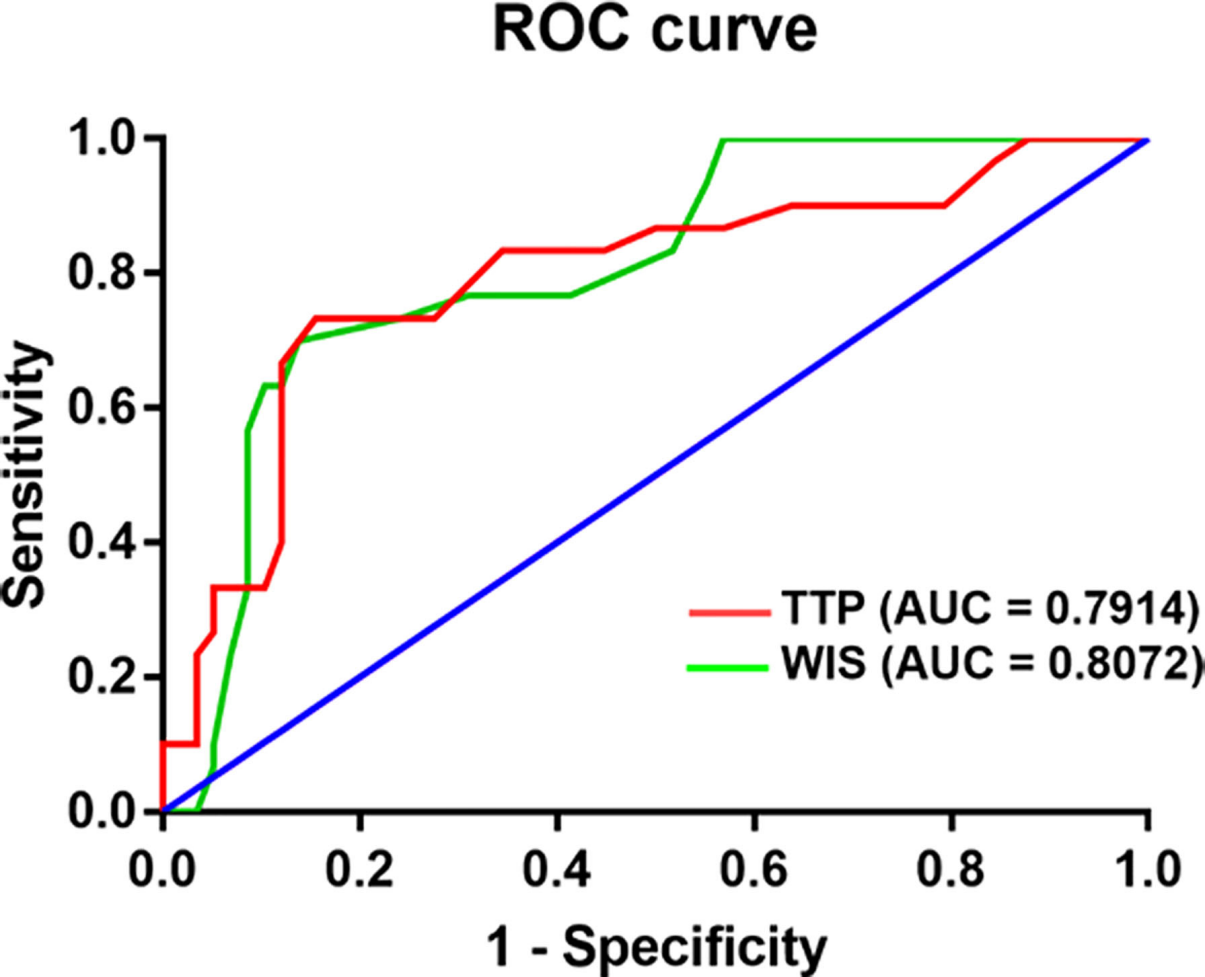
Diagram shows Comparison receiver operating characteristic curve of CEUS between granulomatous lobular mastitis and breast cancer.

For GLM patients, the mean TTP and WIS distributions for the BI-RADS categories are shown. There was a significant difference between BI-RADS 3 and 4 lesions (p< 0.05, 95% CI). There was a negative correlation between BI-RADS scores and TTP (p< 0.01) (**Table 3**). According to ROC curve analysis, the best cut-off value of TTP for distinguishing between GLM and breast cancer was 24.5s, yielding sensitivity and specificity of 73.33% and 84.48%, respectively, for the diagnosis of GLM. The cut-off value of WIS was 0.185dB/s, at which the sensitivity and specificity for diagnosing GLM were 86.21% and 70%. ROC results revealed an area under the curve values (TTP-AUC: 0.791, WSI-AUC: 0.807) (**Figure 4**).

**Table 3:** CEUS-BI-RADS Categories,TTP and WIS Values of Patients with GLM.

		TPP (s)	WIS (dB/s)
CEUS-BI-RADS	3	27.92 ± 7.98	0.16 ± 0.05
	4	26.50 ± 3.89	0.15 ± 0.05

## Discussion

This study aimed to compare the CEUS characteristics of GLM to those of breast cancer. To interpret the CEUS results, we used quantitative and qualitative examination parameters that provide a measurable value for the lesions. In our study, GLM had statistically higher TTP parameters (sensitivity 73.33%, specificity 84.48%) and lower WIS values (sensitivity 86.21%, specificity 70%) than breast cancer. The CEUS adds valuable information to that obtained through the US. Our findings indicated that this approach could help to avoid unnecessary biopsies.

GLM is a rare chronic inflammatory, benign breast disease. It primarily affects women of childbearing age, and its etiology is unknown. It is difficult to diagnose because it can mimic breast cancer clinically and radiologically (1). For the initial assessment of this rare entity, ultrasound and mammography are commonly used imaging modalities. However, the imaging findings on ultrasound and mammography are often inconclusive, making it difficult to distinguish this disease from malignancy using these traditional imaging techniques (2, 10). Dynamic contrast enhancement Magnetic resonance imaging (DCE-MRI) is a powerful tool for detecting breast disease. DCE-MRI parameters of breast cancer were found to be related to the expression of histopathological factors (11, 12). GLM characteristics detected by MRI commonly manifest as heterogeneous enhancing masses, segmental non-mass enhancement (NME), or focal non-massive lesions. The most common MRI finding in GLM patients is NME, which is characterized by heterogeneous and clustered ring enhancement patterns (13, 14). However, access to MRI may be limited, and exams are relatively expensive. Furthermore, patients who are contraindicated for MRI or who cannot tolerate MRI are not candidates for these exams. Our study only performed mammography on patients over 40 years of age who had non-diagnostic images. Four patients with GLM for DCE-MRI, and two of the images show a ring-shaped pattern of non-massive enhancement around the lesion.

Several Multiple irregular hypoechoic masses with multiple tubular extensions are a symptom of GLM (15). Shear wave elastography (SWE) values were significantly higher in breast cancer patients than in GLM patients (16, 17). This study’s most common ultrasound findings were increased skin thickness and irregular heterogeneous hypoechoic masses with tubular extension, which is consistent with previous research. These findings mainly were BI-RADS 4-5 and necessitated a biopsy for a definitive diagnosis (18).

CEUS is currently a widely used diagnostic method for assessing microvascular architecture in real-time. CEUS has an advantage over power Doppler, which has been widely used to assess the vascularity of liver and other organ masses (19). Ultrasound contrast agents (UCA) are gas-filled microbubbles (3–10 m diameter) supported by a flexible shell, such as phospholipids or albumin, employed in CEUS. These microbubbles operate as resonant entities in an ultrasonic field, generating nonlinear scattered signals that distinguish blood flow from surrounding tissue (20). In an ultrasound examination, SonoVue is used to enhance the vascular signals. As a result, the breast’s detection, morphology, and flow of microvessels are improved (21, 22). Previous research has found that CEUS has a higher diagnostic performance than the conventional US in distinguishing benign from malignant breast lesions (23). The use of contrast-enhanced ultrasound in conjunction with blood cell analysis improved the diagnostic accuracy of plasma cell mastitis (24). Min Tang demonstrated that CEUS has high diagnostic accuracy in distinguishing benign inflammation from the malignant peripheral pulmonary disease (25).

In the present study, according to the BI-RADS classification, US scores for GLM in this study were predominantly 4. In patients with GLM, the addition of CEUS reduced the score from 4 to 3. The scores, however, did not change in patients with breast cancer. We hypothesize that CEUS will be an effective tool in evaluating GLM with unclear findings on conventional ultrasound to differentiate between categories 3 and 4. As a result, CEUS may reduce the number of tissue core needle biopsies of GLM. In comparison to MRI, CEUS is a relatively simple, quick, and inexpensive method suited to becoming part of the diagnostic algorithm of breast examination prior to biopsy.

In our study, we used the TI curves to look at differences in vascular perfusion kinetics. When compared to breast cancer, GLM was associated with significantly higher TTP values and significantly lower WIS values. These findings can be explained by the earlier and faster onset of breast cancer enhancement. Several other studies that focused solely on CEUS characteristics of confirmed breast cancer found earlier peak enhancement (analogous to TTP and WIS parameters) and faster microbubble elimination in more aggressive forms of cancer associated with poor prognosis (7, 26, 27). In our research, evaluating other qualitative (vascularization type, perfusion homogeneity) and quantitative parameters (PI, AUC) did not significantly improve the ability to distinguish between GLM and breast cancer. Thus, quantitative CEUS analysis provides an objective and reproducible assessment of lesion vascularization, whereas some quantitative parameters between GLM and breast cancer still overlap. One possible explanation for this is that some hypervascular GLM lesions, particularly at the chronic inflammation stage, mimic malignant tumors because their enhancement dynamics are similar to those of carcinomas.

Sonovue was the most widely used UCA in previous studies, but its mechanical properties limit its use with high-frequency linear array probes for breast scanning and its capacity for long-term imaging. In our study, Sonovue imaging could only last 4 minutes, limiting access to high-quality imaging parameters compared to the more stable Optison or Sonazoid (28). External perfusion software, such as VueBox (Bracco, Italy), with integrated motion correction, allows for a more detailed evaluation of micro vascularization in terms of wash-in and wash-out kinetics because cine loops for up to 2min can be evaluated and more parameters are determined (29). As a result, new UCA in conjunction with analysis software may improve CEUS diagnostic performance.

There are several limitations to our study. First, neither inter nor intra-observer variability was assessed. Second, statistical power may have been compromised because this was a single-center clinical study with a small number of patients. This study was not intended to replace conventional US for the diagnosis of GLM and breast cancer but rather to describe how a complementary method, based on the microcirculation of the tissues examined, can provide valuable additional information to that obtained using the US.

## Conclusion

According to the findings of this study, CEUS has a favorable diagnostic performance with a higher TTP and a lower WIS value in distinguishing GLM from breast cancer. Applying this method in clinical practice can influence clinical decision-making for further biopsies.

## Data Availability Statement

The original contributions presented in the study are included in the article/supplementary material. Further inquiries can be directed to the corresponding author.

## Ethics Statement

The studies involving human participants were reviewed and approved by Ethics Committee of Jiangsu University Affiliated People’s Hospital. Written informed consent for participation was not required for this study in accordance with the national legislation and the institutional requirements. Written informed consent was obtained from the individual(s) for the publication of any potentially identifiable images or data included in this article.

## Author Contributions

LY, AA, and QZ contributed to the conception and design of the study. XX organized the database. LP performed the statistical analysis. LY wrote the first draft of the manuscript. TW, AA, and XQ wrote sections of the manuscript. AA and XQ revised the manuscript. All authors contributed to the article and approved the submitted version.

## Funding

This study was supported Research Project of Jiangsu Maternal and Child Health Association (Grant No. FYX202004) and Zhenjiang Social Development Guidance Project (Grant NO. FZ2018035).

## Conflict of Interest

The authors of this manuscript declare no relationships with any companies whose products or services may be related to the subject matter of the article.

## Publisher’s Note

All claims expressed in this article are solely those of the authors and do not necessarily represent those of their affiliated organizations, or those of the publisher, the editors and the reviewers. Any product that may be evaluated in this article, or claim that may be made by its manufacturer, is not guaranteed or endorsed by the publisher.
